# Essential Oils and Health

**Published:** 2020-06-29

**Authors:** J. Tyler Ramsey, B. Carrie Shropshire, Tibor R. Nagy, Kevin D. Chambers, Yin Li, Kenneth S. Korach

**Affiliations:** a Campbell University School of Osteopathic Medicine, Lillington, NC; b Reproductive and Developmental Biology Laboratory, National Institute of Environmental Health Sciences, National Institutes of Health, Research Triangle Park, NC

**Keywords:** Endocrine disruptors, prepubertal, gynecomastia, psychological, antimicrobial, anti-inflammatory

## Abstract

Essential oils (EOs) have risen in popularity over the past decade. These oils function in society as holistic integrative modalities to traditional medicinal treatments, where many Americans substitute EOs in place of other prescribed medications. EOs are found in a multitude of products including food flavoring, soaps, lotions, shampoos, hair styling products, cologne, laundry detergents, and even insect repellents. EOs are complex substances comprised of hundreds of components that can vary greatly in their composition depending upon the extraction process by the producer or the origin of the plant. Thus, making it difficult to determine which pathways in the body are affected. Here, we review the published research that shows the health benefits of EOs as well as some of their adverse effects. In doing so, we show that EOs, as well as some of their individual components, possess antimicrobial, antiviral, antibiotic, anti-inflammatory, and antioxidant properties as well as purported psychogenic effects such as relieving stress, treating depression, and aiding with insomnia. Not only do we show the health benefits of using EOs, but we also indicate risks associated with their use such as their endocrine disrupting properties leading to the induction of premature breast growth in young adolescents. Taken together, there are many positive and potentially negative risks to human health associated with EOs, which make it important to bring awareness to all their known effects on the human body.

## Introduction

The essential oil (EO) industry developed into a highly active and successful market over the past decade [1]. Many individuals use essential oil containing commodities regularly, including food flavoring, soaps, lotions, shampoos, hair-styling products, cologne, and laundry detergents [2]. Many people seems to deem essential oils as safe alternatives to more invasive pharmacological forms of treatment due to the concept that they are more “natural.” However, only a modest amount of research has been conducted on essential oils. This leaves the potential beneficial and/or adverse effects unclear, making it necessary to investigate these oils in order to verify their true effects on human health.

There are many methods by which EO exposure can occur including inhalation, ingestion, massage, and skin applications, [3,4]. EOs are known for many of their health effects such as their antibacterial, antibiotic, and antiviral properties [3,5-9]. They are also known for relieving stress and have been used in multiple treatments such as sleep disorders, Alzheimer disease, cardiovascular issues, cancer, and labor pain in pregnancy [3,5-12]. Furthermore, they are also known for their insect repellent properties and antioxidant/anti-inflammatory activity [11,13-15]. Most essential oils are generally safe. The majority of adverse effects are mild, but there have been cases of serious toxic reactions including abortions and pregnancy abnormalities, neurotoxicity, bronchial hyperactivity, hepatotoxicity, prepubertal gynecomastia, and premature thelarche [16-19].

EOs are complex substances, comprised of multi-component mixtures that contains hundreds of chemicals. The oils are typically extracted by steam distillation of plant material [2,20-22]. In an individual oil, up to 400 substances can be identified, or even more when the finest analytical equipment is utilized [20]. In a publication of *Contact Dermatitis*, 4350 chemicals were found in 91 EOs [23]. The composition of an EO can vary considerably between producers as well as between the same producer. Many of the factors that can change an EO chemical composition includes the species, origin, climate, soil conditions, fertilization, and mode of production. Terpenes are the biggest class of chemicals found in essential oils. This group of chemicals are created from 5-carbon isoprene units. Larger, more sophisticated molecules, can be constructed in biosynthesis from terpenes to make linear-chained chemicals with one or more ring structures [20]. There are several classes of terpenes, however, the most important in essential oils are the monoterpenes and sesquiterpenes [20]. The distinct smell of an EO is produced from these two groups of chemicals. Modification of a terpene or sesquiterpenes, typically from oxidation or rearranging the skeletal structure of the molecule, yields different terpenoids. The oxidation reactions are most important, which create many subgroups such as alcohols, aldehydes, phenols, ethers, and ketones [20,24,25]. Thus, these oils are widely variable in their composition and make it difficult to assess the health effects each time they may be used.

## Endocrine Disrupting Activities

According to the United States Environmental Protection Agency, an endocrine disrupting chemical (EDC) is an exogenous agent that interferes with the production, release, transport, metabolism, binding, action, or elimination of natural hormones in the body responsible for the maintenance of homeostasis and the regulation of developmental processes [26,27]. An EDC may interfere with hormone action by several mechanisms and can be quite complex. The chemicals may bind to hormone receptors and act directly as an agonist or antagonist, exert indirect agonist or antagonist actions, or may bind to allosteric sites and yield unanticipated effects at very low concentrations [28]. In addition, these chemicals are known to interfere with hormone synthesis, metabolism, transport, and degradation [28].

In previous reports, essential oils have been determined to act as an EDC [16-18]. Essential oils have been demonstrated to act as an agonist to the estrogen receptor alpha (ERα) and antagonist to the androgen receptor (AR) [16-18]. Additionally, these studies have provided support to a suspected link between abnormal breast growth in adolescents, termed prepubertal gynecomastia and premature thelarche, and regular topical exposure to lavender or tea tree oil hygiene commodities [16-18]. Premature thelarche, the most common pubertal disorder in prepubescent girls, which is defined as isolated breast growth before 8 years of age without any other signs of puberty.

Gynecomastia is suspected to have many etiologies. Selected drugs and environmental exposures such as alcohol, heroin, marijuana, amphetamines, antiulcer medications, antibiotics, cancer agents, cardiovascular drugs, and psychoactive drugs have been identified as possible hormonal mimics for the estrogen and androgen receptors [16-18]. The mechanism by which those drugs disrupt the endocrine system is poorly defined but could also involve altering steroidogenesis, with a resultant change in the balance between testosterone and estradiol (E2) levels, increasing proliferation of breast tissue and leading to the onset of gynecomastia [17,29].

**Figure 1:**
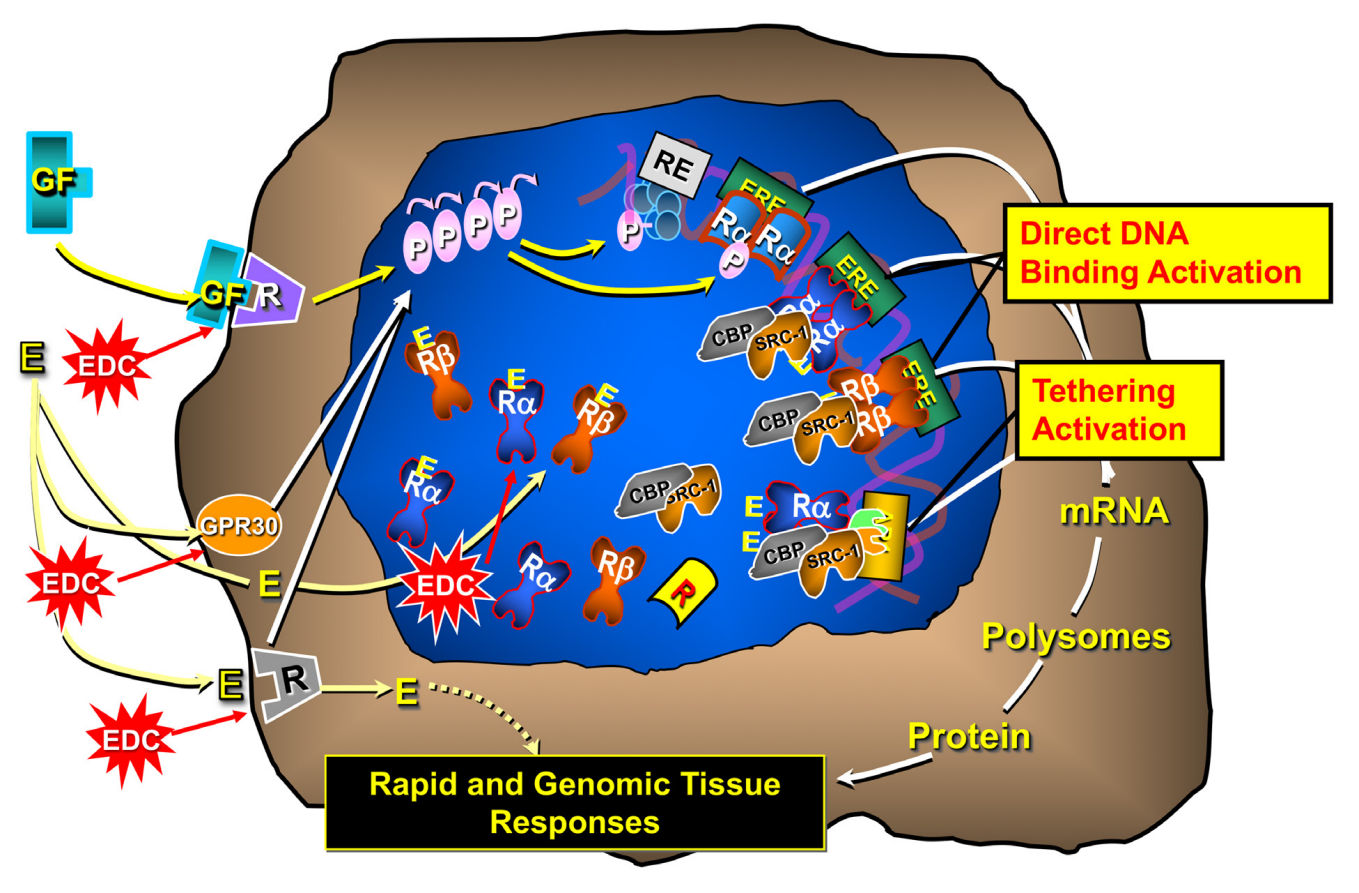
Proposed Mechanism for EO and components Agonizing and Antagonizing the ERα and/or AR Receptor mechanisms. Estrogen or androgen hormones can elicit biological responses by interaction with cell membrane-based receptor proteins (GPR30 or R) to instigate intracellular agonist signaling mechanisms. The hormones can stimulate agonist activities by interacting with nuclear forms of the receptor proteins to stimulate DNA binding genomic mechanisms of gene regulation (direct or tethering). Nuclear hormone receptors can also be activated in a ligand independent mechanism by other intracellular signaling mechanisms (*e.g.* Growth Factors). EO acting as endocrine disruptors can alter any of these possible cellular mechanisms.

Some EDCs act through nuclear hormonal receptors, while others initiate their effects through different mechanisms [30]. Previous studies have reported that ERα plays a crucial role in mammary gland development using knockout (KO) mouse models. In both aromatase KO mice that lack endogenous estrogen production and ERα knockout mice that lack functional ERα, impaired mammary gland development was exhibited [31]. This supports the view that estrogen-dependent, ERα-mediated actions are critical for mammary gland development and could be the reason for these observations seen in the prepubertal children [32-35]. Figure 1 demonstrates the proposed cellular mechanism of action in which these essential oils produce their biological effects on the human body.

## Antimicrobial, Antiviral, and Antibiotic Effects

Essential oils are common natural products that can be used for various medical applications, and in combination with the emergence of antimicrobial resistance, essential oils have been studied as potential antimicrobials agents [36]. These naturally occurring compounds are linked to having bactericidal, virucidal, and fungicidal activity in clinical trials. It has also been suggested that these plant extracts might not only be used to fight cutaneous infections for example, but also serve a role in the preservation of food due to their antimicrobial activity combined with their antioxidant property [36,37]. Table 1 provides a brief summary of certain common essential oils and the organisms targeted.

Bacterial infections remain a significant cause of mortality in the human population. This has triggered research into the exploration of alternative therapies against bacterial strains as the issue of antibiotic resistance has become more imminent even to the newest antibiotic drugs. The effect of antibacterial activity of essential oils may be bacteriostatic or bactericidal, but is difficult to distinguish these actions therefore activity is commonly measured as the minimum bactericidal concentration (MBC) or the minimum inhibitory concentration (MIC) [38,39]. The mechanism of antibacterial action is facilitated by a succession of biochemical reactions within the bacterial cell that are dependent on the type of chemical constituents present in the essential oil. Due to these compounds being lipophilic, essential oils easily penetrate bacterial cell membranes and have been reported to disrupt critical processes of the cell membrane like nutrient processing, synthesis of structural molecules, emission of growth regulators, energy generation, and influences on the cell-cell communication quorum sensing network [4,39,40]. The list of specific bacteria targeted by the essential oils is expanding and include, but are not limited to, *Listeria monocytogenes, Bacillus sphaericus, Enterobacter aerogenes, Escherichia coli O157:H7, P. aeruginosa, S. aureus, S. epidermidis, S. typhi, Shiguella flexneri,* and *Yersinia enterocolitica* [41-44]*. *Some of the essential oils commonly used come from garlic, ginger, clove, black pepper, green chile, cinnamon, clove, pimento, thyme, oregano, and rosemary [39].

Similarly to the effects on bacteria, essential oils have the ability to enter and interrupt the homeostasis of the fungal cell wall and cytoplasmic membranes, specifically the mitochondria [37,39,45]. One of the mechanisms suggested involves the penetration of essential oils into the mitochondrial membranes and changing the electron flow through the electron transport system, which in return disrupts the lipids, proteins, and nucleic acid contents of the fungal cells [46]. Another proposed mechanism is the depolarization of the mitochondrial membranes that decreases the membrane potential, affecting ion channels to reduce the pH and affect the proton pump leading to fungal cell apoptosis and necrosis [47]. Extracts from plants such as basil, clove, citrus, garlic, fennel, lemongrass, oregano, rosemary, and thyme have demonstrated their significant antifungal activity against a broad range of fungal human pathogens [48]. Some of the fungal pathogens affected include *Candida acutus, C. albicans, C. apicola, C. catenulata, C. inconspicua, C. tropicalis, Rhodotorula rubra, Sacharomyces cerevisae, *and *Trignopsis variabilis, Aspergillus parasiticus,* and *Fusarium moniliforme* [39,41,49].

Since viral infections are still a problem for human health and only a narrow number of drugs are effective, it has prompted researchers to explore new antiviral molecules that can attack these human pathological viruses. Detailed insight on the antiviral action of essential oils still requires more research. Essential oils might interfere with virion envelopment, which is designed for entry into human host cells, synthesis of viral proteins, inhibition of the early gene expression process, glycosylation process of viral proteins, and inhibition of virus replication by hindering cellular DNA polymerase [50-53]. Some of the pathogens targeted include many DNA and RNA viruses, such as herpes simplex virus type 1 (HSV-1) and type 2 (HSV-2), dengue virus type 2, Junin virus, influenza virus adenovirus type 3, poliovirus, rhinovirus, and coxsackievirus B1 [39]. Activities of essential oils extracted from Australian tea tree oil, eucalyptus oil, thyme oil, and many other medicinal and aromatic plants have been studied for their effect against viruses [39,45].

## Insect Repelling Properties

Arthropod borne infectious diseases are found in zoonotic reservoirs such as birds and mammals. They are transmitted to humans via the bite of infected mosquitoes, midges, flies, fleas, and ticks [54]. Currently, there are few vaccinations available to prevent the transmission of arthropod borne infectious diseases; human transmission prevention relies on arthropod avoidance, insect repellents, and insecticides [54]. Consequently, development of safe and effective therapies against arthropod borne diseases is of utmost importance.

Insect repellents may be synthetic or organic and discourage insects from contact or biting [55]. Among the most commonly used repellents are synthetic repellents such as DEET (N,N-diethyl-3- methylbenzamide, formerly N,N-diethyl-m-toluamide), which has recently raised concerns relating to its environmental, human health, and safety risks [11,56]. Thus, consumers are apprehensive of its use as well as the use of other synthetic repellents [49]. Therefore, plant EOs have been considered as an organic alternative to synthetic repellents such as DEET due to their improved safety and toxicity profiles to humans and the environment [11,57,58]. A comprehensive list of plant EOs exhibiting arthropod repellent properties may be found below in Table 2 [57].

The components of EOs that have been shown to give them repellent activity are monoterpenoids, sesquiterpenes, and alcohols [11,13,59]. Monoterpene repellent compounds include a-pinene, cineole, eugenol, limonene, terpinolene, citronellol, citronellal, camphor, and thymol [57,60-63]. β-caryophyllene is a sesquiterpene with repellent activity [57,64]. Phytol, phenylethyl alcohol, β-citronellol, cinnamyl alcohol, geraniol, and α-pinene are all alcohols with strong repellent activity [57,65]. These constituents have shown repellent activity against mosquitoes, specifically *Aedes aegypti *and* Anopheles gambiae*, as well as ticks including *Ixodes ricinus *[57]. The combination of EOs from different plants is believed to lead to a synergistic activity, increasing the effectiveness of EOs as insect repellents when compared to individually isolated components [11]. This synergistic phenomenon has been observed when combining monoterpenes with sesquiterpenes [11,66]. Some EO plant combinations lead to a decrease in activity when compared to their individual use. This emphasizes the importance of examining and researching the minor constituents of EOs and their effect on repellency [11].

There are a multitude of plant EOs with repellent properties as seen in Table 2. EOs are highly volatile compounds that exert their activity while in their vapor phase [11,67,68]; meaning their activity typically does not last long requiring frequent reapplication for a short protection time [11]. Scientists are currently developing means to retain the active components on the skin for longer periods of time [11]. Some current advances increasing repellency duration include cream-based formulations, polymer mixtures, microencapsulated extended release, fixative agents like vanillin, nanoparticle fabrication, and polymeric repellent patches [11,15,69,70].

Due to the above-mentioned use, the main concern regarding safety and toxicity of plant EOs is skin irritation. Other negative side effects noted have been asthma, contact dermatitis, headache, increased bleeding, eye-irritation, neurotoxicity, genotoxicity, and immunotoxicity [11]. Citronella use has been banned in Europe and Canada since 2006 due to lack of safety information and the presence of methyl eugenol [11]. Methyl eugenol has shown carcinogenic traits in animal studies with no data available in human studies [11,71]. The US National Toxicology Program did state that methyl eugenol is “reasonably anticipated to be a human carcinogen” [11]. Clove oil also contains methyl eugenol and has yet to be evaluated for carcinogenic properties. It is used not only in insect repellents, but in food, cosmetics, and medicines as well [11].

Plant EOs as insect repellents are of high interest due to their overall improved safety profile when compared to their synthetic counterparts such as DEET. The synergism observed when combining different plant EOs and the experimentation with things such as extended release formulations are extending the repellent activity of plant EOs. More research should be conducted noting plant EOs minor constituents and their contribution to repellency. Research is lacking in the health risks associated with plant EOs as insect repellents. Plant EOs are overall promising alternatives to synthetic compounds demonstrating a need for increased focus in the field of multiomics for their improvement and development.

## Anti-Inflammation and Antioxidant Properties

Inflammation is the body’s response to noxious stimuli such as infection or tissue injury; the response depends on biological, chemical, and mechanisms [72-74]. EOs such as chamomile, eucalyptus, rosemary, lavender, millefolia, have been found to mediate the inflammatory response [14]; they have the ability to influence antioxidant activity, signaling cascades, cytokines, regulatory transcription factors, and the expression of pro-inflammatory genes [14]. The three main anti-inflammation properties of EOs include inhibition of arachidonic metabolism, cytokine production, and pro-inflammatory gene expression [14].

**Figure 2:**
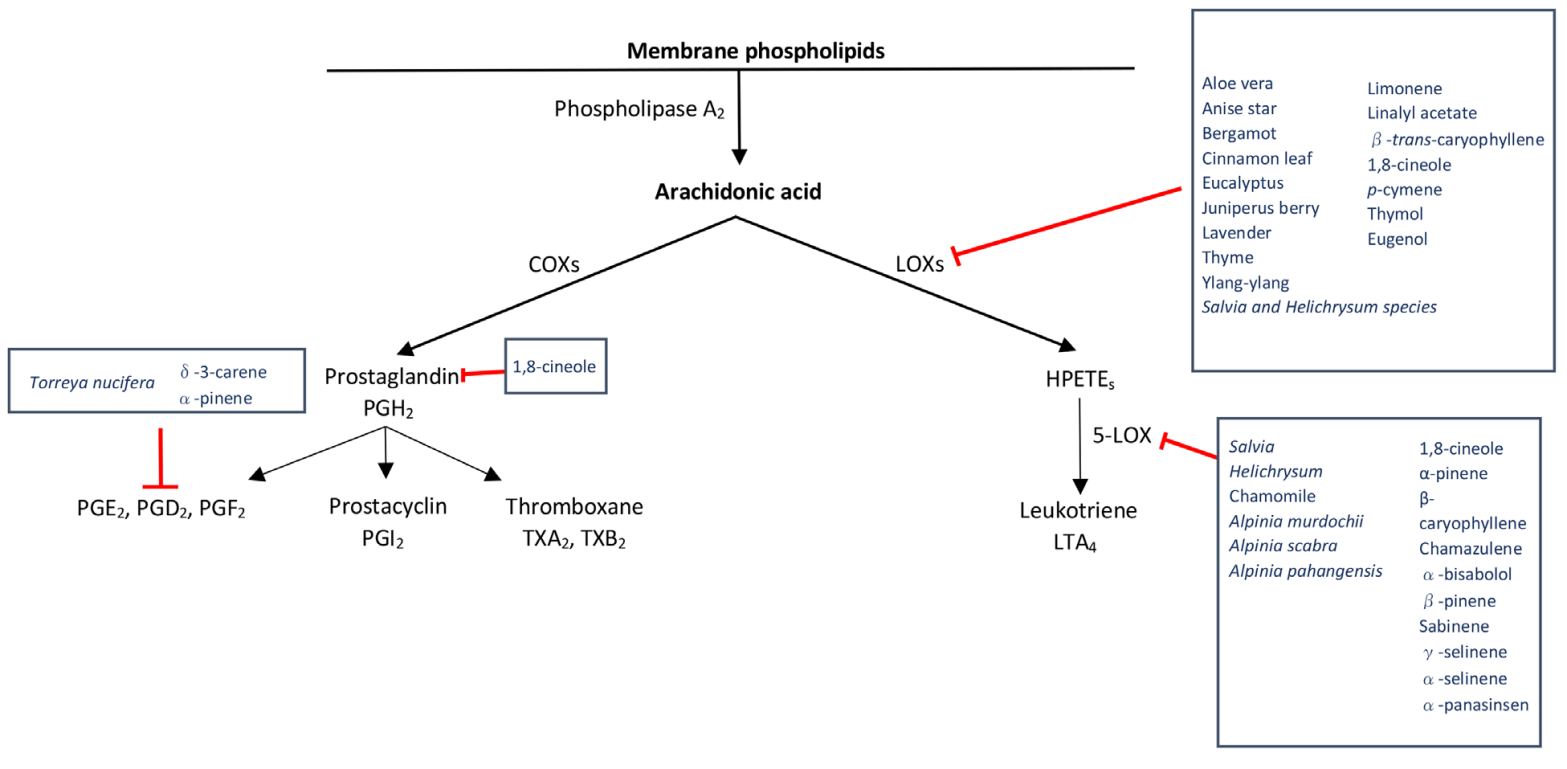
Schematic representation of the inhibition of arachidonic acid metabolism via EOs and their constituents. COX= cyclooxygenase, LOX= lipoxygenase, HPETE= hydroperoxyeicosatetraenoic acid. Figure adapted from Chapter 7, Pathogenesis and Progression of Multiple Sclerosis: The Role of Arachidonic Acid–Mediated Neuroinflammation [118].

Arachidonic acid is released by the cell membrane via phospholipase A_2_ as part of the inflammatory response and further metabolized through either the cyclooxygenase (COX) or lipoxygenase (LOX) pathway [14]. The COX pathway produces prostaglandins (PGs) and thromboxane A_2_ while the LOX pathway produces leukotrienes (LTs) [14]. Inhibiting either pathway leads to a reduction in inflammation via reduction of PGs, thromboxane A_2,_ and LTs, key inflammatory mediators. Aloe vera, anise star, bergamot, cinnamon leaf, eucalyptus, juniperus berry, lavender*, *thyme, and ylang-ylang, are all EOs containing limonene, linalyl acetate, β-*trans*-caryophyllene, 1,8-cineole, *p*-cymene, thymol, and eugenol which inhibit the LOX pathway [14,75]. EOs of the *Salvia and Helichrysum species *express 1,8-cineole, α-pinene and β-caryophyllene inhibiting 5-lipoxegenase [14,76,77]. Chamomile’s constituents chamazulene and α-bisabolol inhibit 5-lipoxegenase [14,78]. *Alpinia murdochii*, *Alpinia scabra,* and *Alpinia pahangensis* also inhibit 5-lipoxynease via their main components β-pinene, α-pinene, sabinene, γ-selinene, α-selinene, and α-panasinsen [14,79]. A common component of EOs, 1,8-Cineole, inhibits both LTs and PGs affecting both pathways of arachidonic acid metabolism [14,80]. *Torreya nucifera* contains δ-3-carene and α-pinene, selectively inhibiting the COX-2 pathway and PGE2 production [14,81]. Figure 2 organizes and summarizes EOs and their components effects on arachidonic acid metabolism.

The innate and adaptive immune response generates cytokines; cytokines play a major role in immune and inflammatory processes of the body [82]. Significant pro-inflammatory cytokines include interleukin-1β (IL-1β), tumor necrosis factor-α (TNF-α), IL-6, and IL-8 [14]. Cytokine signaling via lipopolysaccharide (LPS) found on Gram-negative bacterial cell walls, lipoteichoic acid (LTA) found on Gram-positive cell walls, and peptidoglycan leads to inflammation, tissue destruction, and loss of function [14,83,84]. EOs inhibit the (LPS)-induced secretion of IL-1β and TNF-α, including *Cheistocalyx operculatus *[14,85]. Tea tree oil’s main constituent terpinen-4-ol prevents the production of cytokines TNF-α, IL-1β, IL-8, IL-10, and PGE2 via LPS [14,86]. Tea tree oil also prevents production of pro-inflammatory cytokine IL-2 while increasing the production of anti-inflammatory cytokines IL-4 and IL-10 [14]. *Taxandria fragrans *components 1,8-cineole, α-pinene, and linalool inhibit TNF-α and IL-6 [14,87]. Components of the EO *Cinnamomum osmophloeum* 1,8-cineole, santoline, spathulenol and caryophyllene oxide decrease production of IL-1β and IL-6 [14]*. Rosmarinus officinalis *EOs constituents 1,8-cineole*,* α-pinene, camphor, and *p*-cymene inhibit IL-6 production [14,88]. *Cinnamomum osmophloeum* EO contains cinnamaldehyde which obstructs IL-1β and TNF-α production [14]. *Cordia verbenacea *EO reduces TNF-α levels via components such as (-)-*trans* caryophyllene and α-humelene. IL-1β levels reduce TNF-α levels and are also affected by α-humelene [14,89-91]. *Cryptomeria japonica *oil inhibits IL-1β, IL-6, and TNF-α with components kaurene, elemol, γ-eudesmol, and sabinene [14,92]. *Artemisia fukudo *and its constituents α-Thujone, β-thujone, camphor, and caryophyllene inhibits TNF-α, IL-1β, and IL-6 [75]. Both eugenol from *Syzygium aromaticum* and citral from *Cymbopogon citratus* decrease secretion of IL-1β and IL-6 [14]. Eugenol also prevents secretion of TNF-α and PGE_2_ [14]. *Cinnamomum insularimontanum *inhibits TNF-α through the action of citral. Myristicin from nutmeg oil inhibits TNF-α release [14]. *Pterodon emarginatus* oil contains *trans*-Caryophyllene, β-elemene, and germacrene reducing IL-1 and TNF-α levels. Thyme containing *p*-cymene and thymol and oregano containing carvacrol inhibit IL-1β and IL-6 [93]. All of the above mentioned EOs and EO constituents act as antagonists to pro-inflammatory cytokine activity. A summary of those constituents and their activities may be found in Table 3.

Nitric oxide (NO) is a free radical produced either enzymatically or non-enzymatically. Enzymatic production of NO via NO synthase is a redox reaction that breaks down L-arginine to L-citrulline and NO; the reaction requires oxygen and NADPH [94]. Non-enzymatically, NO is produced from nitrite under acidic conditions such as ischemia [94]. NO modulates the inflammatory response by regulating transcription factors such as NF-κB, AP-1, Jak-STAT, bacterial transcription factors, in addition to monitoring the levels of neutrophils, and eosinophils [94]. EOs *Teucrium brevifolia* and *Teucrium montbretia *directly inhibit NO production thus inhibiting the inflammatory response; spathulenol, δ-cadinene, carvacrol, 4-vinyl guaiacol, and caryophyllene oxide are their constituents [14]. *Fortunella japonica *and *Citrus sunki, *both containing limonene, also inhibit NO production and inflammation [14]. *Origanum ehrenbergii *oil with thymol and *p-*cymene exhibits NO inhibition, along with citrus peel and *Distichoselinum tenuifolium, *composed of myrcene [95]*. *EOs* Cryptomeria japonica, Abies koreana, Farfugium japonicum, Illicium anisatum, Juniperus oxycedrus, Cinnamomum insularimontanum, *and *Juniperus oxycedrus *and constituents 1-undecene, 1-nonene, β-caryophyllene, 1,8-cineole were all found to inhibit NO production [14]. Regulation of NO and inflammation via inhibition of NF-kB transcription has been observed by EOs including *Pimpinella, Artemisia fukudo, Cleistocalyx operculatus, Juniperus oxycedrus *[14]. Their constituents include α-thujone, β-thujone, camphor, caryophyllene, anethole, eugenol, α-pinene, and isoeugenol [14].

## Psychological Effects

Generalized Anxiety Disorder (GAD) is characterized by persistent and excessive worry with associated psychic and somatic symptoms [96]. GAD is a common condition that can lead to significant personal and social impairment [97]. Current treatment modalities for GAD include cognitive behavioral therapy, as well as medical therapy primarily with benzodiazepines or antidepressants [98]. Essential oils represent a potential new treatment category for GAD. Animal models have demonstrated anxiolytic properties in certain essential oils including *Lavendula angustifolia*, *Citrus sinensis,* and *Citrus aurantium* subspecies bergamia [99]. These properties have been demonstrated to be replicable in human clinical trials [100-102]. The method of administration also appears to play a role in the effectiveness of these products, with the three most common administration routes being inhalation, oral, and topical. Anosmia models have been used in experimental animal studies that show the anxiolytic effects of lavender still occurs even if the olfactory receptors have been disabled [103]. Studies are beginning to elucidate the mechanism of action of essential oils. Many essential oils exert their central nervous system pharmacological properties through interactions with serotonin receptors, the GABAergic system, and voltage-gated Na^+^ channels [104]. Inhalation of bergamot (*Citrus bergamia*) oil could regulate the blood pressure and heart rate of healthy volunteers [105]. Lemon essence has been studied in palliative care patients and was shown to increase heart rate, diastolic blood pressure, and respiratory rate in both conscious and unconscious patients, while lavender oil was found to have opposing effects [106]. Interestingly, some essential oils have been associated with worsened anxiety symptoms. Specifically, lemon essence was shown to worsen nociceptive and anxiety responses in rats [107].

One challenge to studying the effects of essential oils has been isolating the active compounds. Harvesting essential oils from their natural reservoirs presents a challenge in ensuring standardization of components as chemical composition can vary based on numerous factors including, geographical location and timing of harvest [108]. In a study of *Satureja* oil, varying chemical compositions were isolated from members of the same genus of plant, which led to significant changes in anxiolytic effects [109]. Another challenge has been the inherent bias present through inhalation methods, as adequate blinding is difficult to achieve due to the recognizable nature of many essential oils. However, oral essential oil products like Silexan have been used in randomized double-blind studies and demonstrated statistically significant anxiolytic activity [110]. Silexan has even been shown to be as effective in reducing anxiety symptoms as paroxetine and lorazepam, with additional improvement in comorbid depression and impaired sleep [111].

Depression is an extremely prevalent mental health disorder characterized by decreased mood, loss of interest, hopelessness, and impaired social function [112]. Traditional antidepressant medication functions through neurotransmitter modulation, but many patients do not experience complete remission of symptoms with monotherapy alone [113]. There have been many studies researching other natural products as alternative antidepressant therapies, specifically St John’s Wart. St John’s Wart is superior to placebo in improving depression symptoms and not significantly different from antidepressant medication [114]. Essential oils represent a potential additional treatment modality for depression [115]. Lavender oil specifically has been shown to ameliorate the depression-like behavior induced by the chronic administration of corticosterone [115].

## Concluding Remarks

EOs have a variety of effects on human health. As it has been demonstrated in many studies, these oils have many psychological effects such as reducing anxiety, treating depression, and even aid with falling asleep. Additionally, they have also been shown to possess antimicrobial, antiviral, antioxidant, anti-inflammatory properties and used as an alternative to synthetic insect repellents. As there are many proven health benefits to essential oils, there are also adverse effects. It has been shown that certain essential oils and their components contain EDCs which appear to have enhanced breast growth in prepubertal children. Taken together, there has been a great amount of research performed in the essential oil field but considering their multitude of components and the spectrum of possible activities there is still a vast amount unknown about their true effects on human health.

## Figures and Tables

**Table 1:** Brief summary of common Essential Oils plant of origin and microorganisms affected by compound extracted. Adapted from [39,45,116].

**Common Name**	**Plant**	**Major Essential Oil**	**Inhibited Microorganisms**
Thyme	*Thymus vulgaris*	Thymol	*S. aureus, V. parahaemolyticus, C. perfringens*
Oregano	*Origanum vulgare*	Carvacrol	*Polio virus, Adeno virus, L. monocytogenes*
Garlic	*Allium sativum*	Isothiocynate	*Candida spp., Enterobacteriaceae*
Lemon Balm	*Melissa officinalis*	Linalool, myrcene, camphor	*HSV-2, avian influenza virus*
Cinnamon	*Cinnamomum zelancium*	Cinnamaldehyde	*Enterobacteriaceae, P. mirabilis, S. pyogenes*
Lavender	*Lavandula angustifolia*	Linalool, Linalyl acetate	*E. coli, M. smegmatis*

**Table 2:** Plant essential oils exhibiting arthropod repellence.

**Essential oil used as repellant**			**Animal species repelled**	
**Plant source**	**Plant family**	**Part used**	**Order**	**Scientific name**
*Mentha piperita*	Lamiaceae	Fresh leaves	Diptera	*Anopheles annularis*
*Mentha piperita*	Lamiaceae	Fresh leaves	Diptera	*Anopheles culicifacies*
*Mentha piperita*	Lamiaceae	Fresh leaves	Diptera	*C. quinquefasciatus*
*Z. piperitum*	Rutaceae	Dried fruits	Diptera	*A. aegypti*
*Pimpinella anisum*	Umbelliferae	Seed	Diptera	*Culex pipiens*
*O. basilicum*	Lamiaceae	Dried foliage	Diptera	*Culex pipiens*
*Eucalyptus camaldulensis*	Mirtaceae	Dried fruits	Diptera	*Culex pipiens*
*Baccharis spartioides*	Compositae	N.I.	Diptera	*A. aegypti*
*Aloysia citriodora*	Verbenaceae	N.I.	Diptera	*A. aegypti*
*Eucalyptus maculate citriodon*	Mirtaceae	Leaves	Diptera	*Mansonia*
*Croton pseudopulchellus*	Annonaceae	N.I.	Diptera	*A. gambiae*
*Mkilua fragrans*	Annonaceae	N.I.	Diptera	*A. gambiae*
*Endostemon tereticaulis*	Labiateae	N.I.	Diptera	*A. gambiae*
*Ocimum forskolei*	Labiateae	N.I.	Diptera	*A. gambiae*
*Ocimum fischeri*	Labiateae	N.I.	Diptera	*A. gambiae*
*Plectranthus longipes*	Labiateae	N.I.	Diptera	*A. gambiae*
*Conyza newii*	Compositae	N.I.	Diptera	*A. gambiae*
*Tarchonanthus camphoratus*	Compositae	N.I.	Diptera	*A. gambiae*
*Tetradenia riparia*	Labiateae	N.I.	Diptera	*A. gambiae*
*Lippia javanica*	Verbenaceae	N.I.	Diptera	*A. gambiae*
*Lippia ukambensis*	Verbenaceae	N.I.	Diptera	*A. gambiae*
*Plectranthus marrubioides*	Labiatae	N.I.	Diptera	*A. gambiae*
*C. citratus*	Poaceae	Fresh aerial parts	Diptera	*A. aegypti*
*O. selloi*	Lamiaceae	Leaves	Diptera	*A. braziliensis*
*O. basilicum*	Lamiaceae	Leaves	Diptera	*Anopheles stephensi*
*O. basilicum*	Lamiaceae	Leaves	Diptera	*A. aegypti*
*O. basilicum*	Lamiaceae	Leaves	Diptera	*C. quinquefasciatus*
*Rosmarinus officinalis*	Lamiaceae	Shoot	Diptera	*Anopheles stephensi*
*Rosmarinus officinalis*	Lamiaceae	Shoot	Diptera	*A. aegypti*
*Rosmarinus officinalis*	Lamiaceae	Shoot	Diptera	*C. quinquefasciatus*
*Cinnamomum zeylanicum*	Lauraceae	Bark	Diptera	*Anopheles stephensi*
*Cinnamomum zeylanicum*	Lauraceae	Bark	Diptera	*A. aegypti*
*Cinnamomum zeylanicum*	Lauraceae	Bark	Diptera	*C. quinquefasciatus*
*C. citratus*	Graminae	N.I.	Diptera	*Culex. quinquefasciatus*
*Zingiber officinalis*	Zingiberaceae	Rhizomes	Diptera	*C. quinquefasciatus*
*Moschosma polystachyum*	Lamiaceae	Fresh leaves	Diptera	*C. quinquefasciatus*
*Solanum xanthocarpum*	Solanaceae	Fresh leaves	Diptera	*C. quinquefasciatus*
*Curcuma longa L.*	Zingiberaceae	Rhizomes	Diptera	*A. dirus*
*C. winterianus*	Poaceae	Leaves	Diptera	*Cx. quinquefasciatus*
*O. americanum*	Lamiaceae	Leaves	Diptera	*Cx. quinquefasciatus*
*Z. limonella*	Rutaceae	Leaves	Diptera	*C. quinquefasciatus*
*Z. limonella*	Rutaceae	Leaves	Diptera	*A. dirus*
*Pogostemon cablin*	Lamiaceae	Commercial	Diptera	*A. aegypti*
*Pogostemon cablin*	Lamiaceae	Commercial	Diptera	*C. quinquefasciatus*
*Pogostemon cablin*	Lamiaceae	Commercial	Diptera	*A. dirus*
*Syzygium aromaticum*	Myrtaceae	Commercial	Diptera	*A. aegypti*
*Syzygium aromaticum*	Myrtaceae	Commercial	Diptera	*C. quinquefasciatus*
*Syzygium aromaticum*	Myrtaceae	Commercial	Diptera	*A. dirus*
*Z. limonella*	Rutaceae	Leaves	Diptera	*A. aegypti*
*C. nardus*	Poaceae	Leaves	Diptera	*A. aegypti*
*E. globulus*	Myrtaceae	Commercial product	Diptera	*A. albopictus*
*D. caryophyllum*	Caryophyllaceae	Flowers	Diptera	*A. aegypti*
*D. caryophyllum*	Caryophyllaceae	Flowers	Ixodida	*A. aegypti*
*Nigella sativa*	Ranunculaceae	Dried fruits	Coleoptera	*T. castaneum*
*Trachyspermum ammi*	Umbelliferae	Dried fruits	Coleoptera	*T. castaneum*
*Anethum graveolens*	Umbelliferae	Dried fruits	Coleoptera	*T. castaneum*
*B. salicifolia*	Asteraceae	Aerial parts	Coleoptera	*T. castaneum*
*Artemisia annua*	Asteraceae	Root	Coleoptera	*T. castaneum*
*Perilla frutesncens*	Labiatae	Leaves	Coleoptera	*L. serricorne*
*Thymus vulagris*	Labiatae	Leaves, Flower, and Stems	Coleoptera	*L. serricorne*
*Satureia hortensis*	Labiatae	Spike	Coleoptera	*L. serricorne*
*Mentha piperita*	Labiatae	Leaves	Coleoptera	*L. serricorne*
*Cinnamomum cassia*	Lauraceae	Bark	Coleoptera	*L. serricorne*
*Litsea cubeba*	Lauraceae	Fruit	Coleoptera	*L. serricorne*
*Perilla frutesncens*	Labiatae	Leaves	Coleoptera	*L. serricorne*
*Laurus nobilis*	Lauraceae	Immature fruits	Coleoptera	*Acanthoscelides obtectus*
*Rosmarinus officinalis*	Labiatae	Flowering shoots	Coleoptera	*Acanthoscelides obtectus*
*E. globulus*	Myrtaceae	Leaves	Coleoptera	*Acanthoscelides obtectus*
*Juniperus oxycedrus*	Cupressaceae	Leaves	Coleoptera	*Acanthoscelides obtectus*
*Lavandula hybrida*	Labiatae	Whole flowering plants	Coleoptera	*Acanthoscelides obtectus*
*Mentha microphylla*	Labiatae	Whole flowering plants	Coleoptera	*Acanthoscelides obtectus*
*Mentha viridis*	Labiatae	Whole flowering plants	Coleoptera	*Acanthoscelides obtectus*
*Apium graveolens*	Umbelliferae	Stems and leaves	Coleoptera	*Acanthoscelides obtectus*
*O. basilicum*	Lamiaceae	Fresh leaves	Coleoptera	*Callosobruchus maculatus*
*Artemisia vulgaris*	Asteraceae	Fresh leaves	Coleoptera	*T. castaneum*
*Ruta graveolens*	Rutaceae	N.I.	Lepidoptera	*C. pomonella (larvae)*
*Allium sativum*	Alliaceae	N.I.	Lepidoptera	*C. pomonella (larvae)*
*Pogostemom cablin*	Laminaceae	N.I.	Lepidoptera	*C. pomonella (larvae)*
*Tanacetum vulgare*	Asteraceae	N.I.	Lepidoptera	*C. pomonella (larvae)*
*Mentha pulegium*	Labiatae	Dried leaves	Phthiraptera	*P. humanus capitis*
*Calocedrus macrolepis*	Cupressaceae	Heartwood	Isoptera	*Coptotermes formosanus*
*Cryptomeria japonica*	Cupressaceae	Sapwood	Isoptera	*Coptotermes formosanus*
*Chamaecyparis obtuse*	Cupressaceae	Leaves	Isoptera	*Coptotermes formosanus*
*Rosmarinus officinalis*	Lamiaceae	N.I.	Thysanoptera	*Thrips tabaci*

N.I. information not available. Table adapted from Luz Stella Nerio; Repellent activity of essential oils: A review [57].

**Table 3:** Representation of essential oils and their constituents that inhibit pro-inflammatory cytokine production.

**Cytokine**	**Main Sources**	**Function**	**Essential Oil Cytokine Inhibition**
IL-1β	Macrophages, monocytes	Pro-inflammation, proliferation, apoptosis, differentiation	*Cheistocalyx operculatus, *tea tree oil, terpinen-4-ol, 1,8-cineole, santoline, spathulenol, caryophyllene oxide, *Cinnamomum osmophloeum, *thyme,* cinnamaldehyde, α-humelene,* thymol,* Cryptomeria japonica, *sabinene*, *citral, kaurene, elemol,γ-eudesmol, eugenol,* α-Thujone, β-thujone, camphor, caryophyllene, Artemisia fukudo, Cymbopogon citratus, *lemongrass,* Syzygium aromaticum*, β- elemene,* Pterodon emarginatus, germacrene, *trans- caryophyllene, *p*-cymene, oregano, carvacrol
IL-6	Macrophages, T-cells, adipocyte	Pro-inflammation, differentiation, cytokine production	*Taxandria fragrans, 1,8-cineole, *thyme, *α-pinene, linalool, santoline, camphor, spathulenol, caryophyllene oxide, Rosmarinus officinalis, p-cymene, Cryptomeria japonica, *sabinene, Citral, kaurene, elemol,γ-eudesmol, eugenol,* α-Thujone, β-thujone, caryophyllene, Artemisia fukudo, Cymbopogon citratus, Syzygium aromaticum, *thymol, *p*-cymene, oregano, carvacrol
IL-8	Macrophages, epithelial cells, endothelial cells	Pro-inflammation, chemotaxis, angiogenesis	Tea tree oil, terpinen-4-ol
TNF-α	Macrophages, NK cells, CD4+ lymphocytes, adipocyte	Pro-inflammation, cytokine production, apoptosis, anti-infection, cell proliferation	*Cheistocalyx operculatus, *tea tree oil, terpinen-4-ol, T*axandria fragrans, *1,8-cineole, α-pinene, linalool, Citral,* Cinnamomum osmophloeum, *eugenol, cinnamaldehyde, *Cordia verbenacea, α-humelene, (-)-trans-caryophyllene, Cryptomeria japonica, sabinene, *kaurene, elemol,γ-eudesmol, α-Thujone, β-thujone, camphor, caryophyllene, *Artemisia fukudo, Cinnamomum insularimontanum, *myristicin, nutmeg oil, germacrene, *Pterodon emarginatus, β- elemene, trans- caryophyllene*

Table adapted from Linlin Chen: Inflammatory responses and inflammation-associated diseases in organs [117].

## Abbreviations

AR: androgen receptor
COX: cyclooxygenase
DEET: N,N-diethyl-3-methylbenzamide, formerly N,N-diethyl-m-toluamide
EDC: endocrine disrupting chemical
EO: essential oil
ERα: estrogen receptor alpha
GAD: generalized anxiety disorder
HSV: herpes simplex virus
IL: interleukin
KO: knock out
LOX: lipoxygenase
LPS: lipopolysaccharide
LT: leukotrienes
LTA: lipoteichoic acid
MBC: minimum bactericidal concentration
MIC: minimum inhibitory concentration
NO: nitric oxide
PG: prostaglandins
TNF: tumor necrosis factor.
